# Cranial Surgery

**Published:** 1895-01-12

**Authors:** 


### PROGRESS IN SURGERY.

### CRANIAL SURGERY.

**Trephining.**—At the International Congress in Rome Lucas Championnière[1] gave interesting details of the results of 64 cases of trephining. In 54 cases the operation was for disease, or old standing injury, the reasons for operation being: True epilepsy, 14; Jacksonian epilepsy, 12; cerebral symptoms, such as vertigo, headache, buzzing in the head, 6; for various palsies, 22. Seven deaths resulted, and in two life was probably shortened, but in all the fatal cases the condition was very serious before operation. In the remaining ten cases the operation was for recent injury, and the results were highly satisfactory, only three very severe cases being fatal. None of the operations were followed by suppuration. In trephining he used a large crown, enlarging the opening if necessary with bone forceps.

The feeling is gaining ground that in trephining more extensive openings are to be recommended. In many cases in which the lesions were missed during life it was found after death that the removal of a little more bone would have exposed them. A lengthy and instructive discussion on intracranial surgery recently took place in Edinburgh,[2] the leading surgeons and physisians taking part. The full report will be read with interest as many cases were detailed. Leonard[3] describes an improved aseptic trephine, in which the trephine is rotated by means of a sliding bar working along a double raised spiral on the shaft. He claims for it greater speed and the necessity of applying less pressure.

**Craniectomy.**—Jacobi[4] has collected statistics of 33 cases of idiocy or microcephalus in which craniectomy was performed. On these 33 cases 41 operations were performed; 19 patients recovered, and 14 died soon after operation. In eight cases slight improvement resulted, and in only two was there much improvement. Jacobi agrees with Bourneville, that probably the operation tends to cause a reduction and not an increase in the cranial capacity. He thinks that the operation, if justifiable at all, is most likely to prove of benefit in cases of uncomplicated premature ossification of the sutures and fontanellas. At Rome Potemski[5] gave the result of 20 craniectomies performed by him. He is disappointed with them. He considers surgical treatment practically useless in hydrocephalus and microcephalus, and also in ordinary epilepsy. He advocates large openings, and does not think exact localisation necessary. Mynter[6] has operated on five cases of microcephalus without any improvement following.

Bennie[7] records two cases operated on for idiocy not associated with microcephalus. In the one case idiocy followed on meningitis; a few hours after operation death took place with hyperpyrexia. In the other case, where mal-development of the cortex was supposed to be the cause of idiocy, much improvement resulted, the child showing capabilities of education formerly absent.

**Tumours, Removal of.**—Steele[8] has successfully removed a fibro-sarcoma of the cerebrum in a man aged 51. The diagnosis was made from the focal symptoms. During the operation faradization with a double electrode over the tumour tissue produced no muscular response. Pel[9] records a remarkable case of cerebral tumour in which there was an absence of headache, giddiness, nausea, vomiting, and optic neuritis—all symptoms usually more or less present, and without some of which diagnosis is doubtful. The gradual development of paralysis and then of epileptic seizures and mental impairment led to the diagnosis of tumour in this case. On trephining, a large cystic fibroma was found in the motor area, but not involving the brain cortex. It was easily removed, but the patient died soon after from shock. Jalland[10] reports a case of glio-sarcoma of the cerebellum, and Bruno[11] two cases, in one the tumour being in the cerebellum and in the other in the corporo quadrigemina.

What seems to have been a brain cyst, following a fall on the anterior part of the parietal region, is described by C. H. Mayo.[12] The patient, a girl aged 11, was seen by Mayo six weeks after injury. During that time she had been comatose. There was right hemiplegia, and the eyes deviated upwards, the left pupil being larger than the right. In the second week convulsions had been present. On trephining over the left fissure of Rolando, and incising the dura mater, four ounces of clear fluid escaped. The frontal lobe was depressed, but no bony fracture was found. Gradual recovery followed, and at the end of a year she seemed quite well. Anderson[13] reports the case of a man, aged 44, who had been subject to epileptiform attacks for nine years, following a fall on the head. Five inches above the left auditory meatus there was a scar, with depression of bone. The trephine was applied at this site, and on the dura being removed a cyst was found pressing on the brain. The fluid contents were evacuated. A good recovery took place without return of the attacks.

**Cerebral Abscess.**—Professor Macewen[14] read a paper on the pyogenic diseases of the brain at the Congress in Rome. He holds (1) that all abscesses of the brain are formed subsequently to a primary focus of infective disease situated elsewhere, (2) that the chief infective foci are formed in connection with middle ear disease, (3) that abscesses of the brain originating in middle ear disease are generally in direct contact with the primary source of infection, (4) that such abscesses are generally best reached in the first place through the mastoid antrum, (5) that the mastoid antrum is most easily opened through the supramental triangle, from which the whole tegmen antri and tegmen tympani may be exposed, (6) it is necessary to remove the whole infective tract, and (7) after this has been done the skull is trephined over the temporo-sphenoidal lobe of the brain. Much valuable information will be found on this subject in Macewen's work on "Pyogenic Infective Diseases of the Brain and Spinal Cord." Mynter[15] records a case in which an abscess, the size of a hen's egg, near the motor centres, was quite unaccompanied by paralysis. The history of a punctured wound of the skull six weeks before the mental depression and the elevation of temperature, led to the diagnosis of abscess. Trephining, and evacuation of the pus and drainage of the cavity, were followed by an excellent recovery. Williamson[16] reports a case of cerebral abscess in a man aged 24, which he considers was secondary to lung disease. The patieut died suddenly, and at the post-mortem examination an abscess was found in the right hemisphere. There was no ear disease or other apparent cause in the cranium, but in the right lung there were bronchiectatic cavities filled with fœtid pus. Williamson classes this case with a group of cases of cerebral abscess, "in which the abscess is the resultant of pre-existent inflammation in some distant part of the body, but in which there are no symptoms of general pyæmia, and in which pyæmic abscesses are generally found in the brain only." He has collected 38 cases of cerebral abscess secondary to purulent lung disease. Naether found that in 49 cases of gangrene of the lung, 37 of fœtid bronchitis, and 12 of bronchiectasis; cerebral abscess occurred in eight. Scheber[17] has successfully trephined in a case of abscess secondary to otorrhœa.

Moullin[18] describes the following case: A boy, aged fourteen, received a blow over the right mastoid. A few days later an abscess superficial to periosteum was opened. A week afterwards this abscess was reopened, and periosteum incised with relief. Five days later symptoms of cerebral pressure set in. The mastoid was trephined, but nothing abnormal found. Shortly afterwards right optic neuritis appeared, and the trephine was applied over the right temporo-sphenoidal lobe. The dura was healthy, but bulged into wound, and there was no pulsation. Exploration with trocar and canula revealed nothing. Bone was also removed from over cerebellum without result, and then a few drachms of fluid were drawn off from the lateral ventricle through the temporo-sphenoidal lobe, pulsation returning. In twenty-four hours patient died comatose. A very old encysted abscess was found post-mortem in the left temporo-sphenoidal lobe. No other lesion was present. This old abscess was probably roused into activity by the blow.

**Epilepsy.**—Kennedy[19] reports five cases treated successfully by trephining. In only two was there a history of the attacks dating from an injury to the head. McCosh[20] records a case of cessation of epilepsy after trephining and typhoid fever. No improvement followed the trephining. Six months later the child developed typhoid fever, and after the first week of the fever the convulsions ceased, and two months later there had been no return. Mynter[6] has operated on five cases during the last two years, and in four there was great improvement. Anderson[21] trephined a man aged twenty-three, who had suffered for nine years from epilepsy. Marked improvement followed.

**Concussion.**—Amongst the most important cases of concussion are those in which there is no real loss of consciousness at all, *i.e.*, where the patient after the fall or blow feels "queer," and giddy for a moment or two, but soon recovers, and is able to recall all that occurred at the time of the accident. Their importance arises from the fact that in these cases the condition is often overlooked. In many no serious harm follows, but there are some in which neglect to submit to rest and quiet is followed by permanent mental weakness. Bennett[22] lays great stress on the facial expression and concussion in diagnosing concussion in such cases. The patients have an unnatural expression, "with a dazed and almost frightened look." The pulse is markedly compressible; to the fingers laid on it, it seems sufficiently strong and full, but the slightest pressure obliterates it. The patient must be kept in bed till the pulse is free from this peculiarity.

**Lateral Sinus Disease.**—Rupture of the lateral sinus is rare, especially when unaccompanied by fracture of the skull. This condition recently came under the notice of Walton.[23] A youth, aged nineteen, received a blow on the left lower jaw, and became unconscious almost immediately afterwards. He presented signs of hæmorrhage at the base of the brain, and as it seemed probable that the medulla was involved operation was deemed useless. He died on the sixth day. Post-mortem examination revealed the presence of black tarry blood beneath the dura over the occipital lobes, especially in the middle fossa, also in the posterior fossa, and extending into the vertebral canal. No bony fracture was found, but there was a small rent in the lateral sinus. Cleghorn[24] has successfully treated a case of thrombosis of the lateral sinus by trephining and ligature of the internal jugular vein. The patient, age seventeen, had suffered from otorrhœa for four years.

**Trigeminal Neuralgia.**—In operating, Reineking[25] lays great stress on thoroughly following up, extracting, and dissecting out the peripheral muscular and cutaneous branches, and on slowly twisting and stretching the central stump until it gives way. He details a case in which he removed as much as one and a-half inches of some of the peripheral branches. There was no return two years later of the neuralgia. Quénu[26] describes his operation; it does not seem to differ much from that of Hartley. Rose[27] has treated two cases by Braunlosen's operation with satisfactory results so far. Mayo Robson[28] records a case treated by Horsley's operation, and Karewski[29] one by Thiersch's method.

**Gunshot Wounds.**—Cases of penetrating gunshot wounds in which the bullets were extracted from the opposite side during life are rare. In a case of Tefft's[30] Nelaton's probe, when introduced through the wound in the frontal region, showed that the track of the bullet passed right through the brain to the occipital region on the same side. The trephine was applied in the occipital region, and after a careful search the bullet was found in the brain, about one and a-half inches below where it had struck the inside of the occipital bone. Death took place twelve days after the injury. Arcoleo[31] records a spontaneous cure after a penetrating bullet wound of the brain, the paralysis being gone at the end of six weeks. At the Chirurgical Society in Paris[32] gunshot wounds were recently discussed with regard to the question of trephining. Opinion seemed to be fairly equally divided between those who advocate immediate operation in all cases and those who prefer to watch carefully for symptoms of complication before trephining. The latter would seem to be the most reasonable course in most cases. No rigid rules can be laid down, however; each case must be treated on its own merits.

**Oeteoporosis of Cranial Vault.**—A case of this affection is fully described by Wherry.[33]

[1] Lancet, April 7th, 1894, p. 887. [2] Edin. Med. Journ., April, May, June, 1894. [3] Annals of Surgery, May, 1894, p. 604. [4] Brit. Med. Journ., June 9th, 1894, Ep. 89. [5] New York Med. Journ., May 5th, 1894, p. 569. [6] Annals of Surgery, May, 1894, p. 539. [7] Brit. Med. Journ., May 19th, 1894. [8] Therap. Gazette, Mar. 15th, 1894, p. 206. [9] Amer. Journ. of Med. Sc., June, 1894, p. 725. [10] Lancet, Mar. 17th, 1894, p. 671. [11] Brit. Med. Journ., Mar. 17th, 1894, Ep. p. 41. [12] Lancet, April, 28th, 1894, p. 1,085. [13] Brit. Med. Journ., Mar. 24th, 1894, p. 636. [14] Brit. Med. Journ., April 21st, 1894. [15] Annals of Surgery, May, 1894, p. 542. [16] Med. Chron., Mar., 1894, p. 379. [17] Lancet, May 12th, 1894, p. 1,204. [18] Lancet, Mar. 17th, 1894, p. 673. [19] Amer. Med. and Surg. Bulletin, Mar. 15th, 1894, p. 333. [20] Annals of Surgery, May, 1894, p. 615. [21] Brit. Med. Journ., Mar. 24th, 1894, p. 637. [22] Clinical Journ., April 4th, 1894, p. 358. [23] Practitioner, May, 1894, p. 367. [24] Brit. Med. Journ., May 5th, 1894. [25] Brit. Med. Journ., May 12th, 1894, Ep. 74. [26] Med. Week, Feb. 12th, 1894, p. 21. [27] Lancet, Mar. 17th, 1894, p. 666. [28] Therap. Gazette, May, 15th, 1894, p. 351. [29] Med. Week, May 25th, 1894, p. 249. [30] Med. Record, April 21st, 1894, p. 495. [31] Brit. Med. Journ., May 12th, 1894. [32] Med. Week, Feb., 1894. [33] Brit. Med. Journ., June 2nd, 1894, p. 1,188.

